# Piezophilic Phenotype Is Growth Condition Dependent and Correlated with the Regulation of Two Sets of ATPase in Deep-Sea Piezophilic Bacterium *Photobacterium profundum* SS9

**DOI:** 10.3390/microorganisms11030637

**Published:** 2023-03-02

**Authors:** An-Qi Li, Wei-Jia Zhang, Xue-Gong Li, Xu-Chong Bao, Xiao-Qing Qi, Long-Fei Wu, Douglas H. Bartlett

**Affiliations:** 1 Institute of Oceanology, Chinese Academy of Sciences, Qingdao 266071, China; 2 University of Chinese Academy of Sciences, Beijing 101408, China; 3 Laboratory of Deep-Sea Microbial Cell Biology, Institute of Deep-sea Science and Engineering, Chinese Academy of Sciences, Sanya 572000, China; 4 International Associated Laboratory of Evolution and Development of Magnetotactic Multicellular Organisms, CNRS-Marseille/CAS-Sanya, Sanya 572000, China; 5 Institution of Deep-sea Life Sciences, IDSSE-BGI, Hainan Deep-sea Technology Laboratory, Sanya 572000, China; 6 LCB, Aix Marseille University and CNRS, 13402 Marseille, France; 7 Marine Biology Research Division, Scripps Institution of Oceanography, University of California San Diego, La Jolla, CA 92093, USA

**Keywords:** deep-sea bacterium, high hydrostatic pressure, piezophilic, ATPase, energy metabolism

## Abstract

Alteration of respiratory components as a function of pressure is a common strategy developed in deep-sea microorganisms, presumably to adapt to high hydrostatic pressure (HHP). While the electron transport chain and terminal reductases have been extensively studied in deep-sea bacteria, little is known about their adaptations for ATP generation. In this study, we showed that the deep-sea bacterium *Photobacterium profundum* SS9 exhibits a more pronounced piezophilic phenotype when grown in minimal medium supplemented with glucose (MG) than in the routinely used MB2216 complex medium. The intracellular ATP level varied with pressure, but with opposite trends in the two culture media. Between the two ATPase systems encoded in SS9, ATPase-I played a dominant role when cultivated in MB2216, whereas ATPase-II was more abundant in the MG medium, especially at elevated pressure when cells had the lowest ATP level among all conditions tested. Further analyses of the Δ*atpI*, Δ*atpE*1 and Δ*atpE*2 mutants showed that disrupting ATPase-I induced expression of ATPase-II and that the two systems are functionally redundant in MB2216. Collectively, we provide the first examination of the differences and relationships between two ATPase systems in a piezophilic bacterium, and expanded our understanding of the involvement of energy metabolism in pressure adaptation.

## 1. Introduction

High hydrostatic pressure (HHP) is one of the major stresses that microorganisms inhabiting the deep sea need to cope with. It is now known that elevated pressure triggers a series of damaging impacts on shallow-water microbial cells, such as disassembly of complexes and decreased membrane fluidity, and hampers diverse biological processes ranging from flagellar motility to bioluminescence, global gene transcription, metabolism, and reproduction [1,2,3]. Piezophiles are a group of microorganisms that grow better under elevated pressures than atmospheric pressure [4]. Increasing evidence has shown that energy metabolism plays an important role in their adaptation to deep-sea HHP environments, along with changing respiratory components. A large number of genes involved in energy metabolism are regulated by pressure in piezophile *Desulfovibrio hydrothermalis*. In addition, the strain has a higher ATP level and lower ADP/ATP ratio at its optimum pressure of 26 MPa than at lower pressures [5,6]. In some piezophiles, the composition of electron transport chains could be modified depending on pressure. For example, at atmospheric pressure, electrons are transferred through three components before eventually being accepted by cytochrome c oxidase in the piezophilic deep-sea strain *Shewanella violacea* DSS12. However, at elevated pressure condition the bd-type quinol oxidase, which is more piezotolerant than cytochrome c oxidase, accepts electrons from ubiquinone directly [7,8,9]. The deep-sea pressure-resistant bacterium *Shewanella piezotolerans* WP3 encodes two types of nitrate reductase systems: the NAP-α system is more tolerant to HHP and presents only in deep-sea strains, while the NAP-β system has only been found in shallower water species. Li et al. showed that the NAP-β system plays a dominant role in nitrate reduction, but the NAP-α system could also be induced by both substrate and HHP in a deletion mutant of NAP-β system [10,11,12]. A similar relationship has been observed in the two types of DMSO reductase systems identified in strain WP3. They differ in subcellular localization and electron transfer route, and are preferably utilized under different conditions. The type I DMSO reductase is essential for growth under in situ conditions of low temperature and high pressure, while type VI DMSO reductase is more favorable at low temperature or high-pressure conditions [13,14].

The electrochemical ion gradient across the membrane established by the electron transport chain drives the rotation of F-ATPase for ATP synthesis. The bacterial F-ATPase complex is composed of two domains. The F_0_ domain embedded in the membrane consists of subunits ab_2_ and multiple subunits of c that form the c-ring architecture. The F_1_ domain is a water-soluble complex consisting of α_3_β_3_γδε. Translocation of protons across the membrane drives the rotation of the rotor composed of subunits γε and c-ring versus the stator, which subsequently induces conformational changes of subunits β and catalyzes the synthesis of ATP. Genes responsible for the F–ATPase synthesis usually form a conserved operon of *atpBEFHAGDC* that encodes for subunits acbδαγβε, respectively. In some cases, a small gene, designated *atpI,* is found upstream of *atpB*. It functions as a chaperone to mediate the assembly of the c-ring, and is indispensable for the synthesis of Na^+^ F-ATPase [15]. Previous studies showed that the ATPase complex was sensitive to HHP. Moderate hydrostatic pressure induces a conformational change of ATPase and leads to a slightly increased activity by 1.5-fold, while higher hydrostatic pressure disassembles the complex and leads to its inactivation [16,17]. Okuno et al. investigated the influence of HHP on the rotation of ATPase at the single-molecule level and they demonstrated that the rotational rate decreased at elevated pressure, which is possible due to a pressure-sensitive ATP docking process [18]. However, how ATP synthesis has evolved in deep-sea bacteria for HHP adaptation remains uninvestigated.

*Photobacterium profundum* SS9 was isolated from biological samples collected at a depth of 2500 m. It is a moderately piezophilic bacterium with an optimum growth pressure of 28 megapascals (MPa) [19,20]. A complete genome sequence and a well-established genetic manipulation system make it a model strain for the study of bacterial adaptation to the deep-sea environment [21,22,23,24,25]. Transcriptional analysis identified a series of genes involved in amino acid and ion transport, stress response and glycolysis repressed by elevated pressures, while genes involved in amino acid fermentation, anaerobic respiration and degradation of complex carbohydrates are up-regulated by elevated pressures [21,26]. A two-component regulatory system, ToxRS, is found to be a major signaling system. It is believed to sense environmental pressure through conformational changes of the membrane, and transmit the signal into the cytosol and regulate gene expression in a pressure-responsive manner [23,26].

SS9 is a facultative anaerobe that is capable of growing through aerobic respiration, anaerobic respiration and fermentation on diverse substrates [21]. Tamegai et al. investigated the terminal oxidase of SS9 grown at 0.1 MPa and 30 MPa. Their results showed that the content of cytochromes and expression of genes responsible for several terminal oxidases were not affected under these conditions, which is apparently different from the other deep-sea piezophilic bacterium *S. violacea* DSS12 [27]. On the other hand, one out of three isoenzymes of TMAO reductase, which is responsible for TMAO anaerobic respiration, was up-regulated by elevated pressure [21]. While the electron transfer chain and terminal oxidase/reductase have been examined, the final step of respiration, synthesis of ATP driven by the proton gradient, has not been investigated in this model strain.

Genomic analysis showed that *P. profundum* SS9 harbors two copies of F-ATPase-encoding loci [21]. One set is located on chromosome I and encodes the nine subunits of AtpI, a, c, b, δ, α, γ, β and ε, and the other set is located on chromosome II and encodes 8 subunits, including all the genes present in the other locus except that encoding for AtpI. In this study, we set out to determine whether or not functional differences exist between the two ATPase systems, including with regard to HHP adaptation. This was accomplished in part by constructing in-frame deletion mutants of distinct (*atpI*) and conserved (*atpE*1 and *atpE*2) genes, and assessing the impact of the loss of these genes on ATPase gene expression and cellular function.

## 2. Materials and Methods

### 2.1. Bacterial Strains and Growth Conditions

The cultivation of the *P. profundum* SS9R (a rifampin-resistant derivative of SS9 strain) and the mutant strains were carried out at 15 °C with Marine Broth 2216 medium (MB2216 medium) (BD Difco, Franklin Lakes, State of New Jersey, USA) or minimal medium supplemented with 20 mM glucose (MG medium) as the sole carbon source. The minimal medium consisted of artificial seawater [0.48 M NaCl, 0.027 M MgCl_2_·6H_2_O, 0.01 M CaCl_2_·2H_2_O, 9 mM KCl, 2.8 mM MgSO_4_·7H_2_O, 4 mM (NH_4_)_2_SO_4_] supplemented with 0.1 M HEPES (pH7.5), 0.05 mM NaH_2_PO_4_, 1 mL vitamin mixture, and 10 mL trace element [28]. For cultivation of strain SS9R and its derivatives, two to three colonies were inoculated into 3 mL fresh medium, and incubated with shaking at 15 °C in the dark for approximately 22 h. The cultures were diluted 1/100 into fresh medium to an initial OD600 nm of about 0.01, then aliquoted into sterilized pipettes (BRAND, Wertheim, Germany) and sealed with a heat-sealing machine. The pipettes were placed into high-pressure vessels filled with pre-cooled water, and pressure was applied with a water pump. The pressure vessels were incubated at 15 °C [29]. Three pipettes were collected for each sampling point and absorption at 600 nm was measured using spectrophotometry (Agilent Technologies Cary 60 UV-Vis, Agilent Technologies, Santa Clara, California, USA). *Escherichia coli* strains were grown in LB medium at 37 °C with shaking at 160 rpm.

### 2.2. Construction of Gene Deletion Mutant

In-frame deletion of *atpI, atpE*1 *and atpE*2 was performed following standard molecular biology techniques as follows. The upstream and downstream of the flanking region of the target gene with a length of about 1 kb were amplified, ligated by fusion PCR and introduced into suicide vector pRE118. The resulting plasmid was transformed into S17-1 λpir by heat-shock transformation and then transferred into SS9R through bi-parental conjugation. A two-round screening was carried out, the first round screening for the colonies resistant to kanamycin and the second round screening for double-cross transformants that survive with the presence of sucrose. The putative mutant strains were verified by colony PCR and sequencing. The primers used in this study are listed in Table S1.

### 2.3. RNA Extraction and Quantitative Real-Time PCR (qRT-PCR)

The total RNA was isolated using TRIzol reagent (Ambion, Austin, USA) according to the manufacturer’s protocol with slight modification. Briefly, 1 mL of mid-log phase cultures were homogenized in 1 mL of TRIzol, and 200 μL of chloroform was added. After a brief vortex, the samples were centrifuged at 12,000× *g* for 15 min at 4 °C. The upper aqueous phase was transferred to a tube containing an equal volume of isopropanol. The mixtures were thoroughly mixed by vortex and centrifuged at 12,000× *g* for 20 min at 4 °C. The pellet was washed twice with 75% ethanol and then solubilized with RNase-free water. The residual genomic DNA was removed by Recombinant DNase I (TaKaRa, Shiga, Japan) following the user’s manual. Approximately 2 μg RNA was applied to reverse transcription reaction with the PrimeScript^TM^ II 1st Strand cDNA Synthesis Kit (TaKaRa, Shiga, Japan) for the synthesis of cDNA.

Quantitative real-time PCR assays were carried out with Applied Biosystems QuantStudio 3 (ThermoFisher Scientific, Shanghai, China) in reaction mixtures with total volume of 20 μL, containing 10 μL of FastStart Universal SYBR Green Master (Rox) (Roche, Mannheim, Germany), 0.5 μM of primer, and 2 μL cDNA template. The relative expression of the target gene was normalized to the reference gene of Glyceraldehyde-3-phosphate dehydrogenase (G3PDH). Three replications were set for each assay. The primers used for qRT-PCR analyses are listed in Table S1.

The absolute quantification of the copy number of target genes was determined by relating the cycle threshold (Ct) value to a standard curve. The *atpI*, *atpE*1, and *atpE*2 from SS9R were cloned into pMD^TM^19-T Vector (TaKaRa, Shiga, Japan). The recombinant vector was transformed into competent *E. coli* DH5α and confirmed by sequencing. Plasmids carrying *atpI*, *atpE*1 or *atpE*2 were extracted and quantified with spectrophotometer (NanoDrop 2000/2000c Spectrophotometers, Thermo Scientific, Shanghai, China). The statistical analysis of comparisons between two groups was performed using an unpaired-sample *t*-test, a two-tailed *p* < 0.05 was considered significant: *, *p* < 0.05; **, *p* < 0.01; ***, *p* < 0.001.

### 2.4. Quantification of Intracellular ATP

The ATP measurements were performed using the BacTiter-Glo™ Microbial Cell Viability Assay kit (Promega, Madison, USA) according to manufacturer’s instructions. Briefly, 100 μL of bacterial culture cultured to exponential phase was plated in black 96-well microplate (Microtiter Solid Cliniplate, 96-Well, Flat, Universal Binding, Black, Thermo Fisher Scientific^TM^, Shanghai, China). Another 100 μL of ATP detection reagent was added and incubated for 2 min. Luminescence was measured using a multifunctional microplate reader (Varioskan LUX, Thermo Fisher Scientific^TM^, Shanghai, China). Medium without inoculation was set as the blank control. Average value was calculated from triplicates of each sample and control. The luminescence intensity of a sample was calculated by subtracting the average signal of blank controls from the average signal of the sample. The statistical analysis of comparisons between two groups was performed using unpaired-sample *t*-test; a two-tailed *p* < 0.05 was considered significant.

## 3. Results

### 3.1. Organization of Two Sets of ATPase Operons in the Genome of Strain SS9R

**Figure 1:**
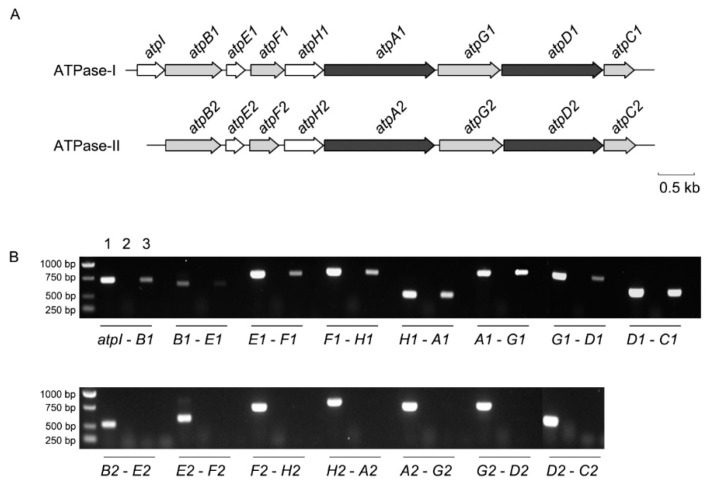
The organization and transcription pattern of two ATPase systems in *P. profundum* SS9R. Panel (**A**), the organization of two ATPase systems in the genome of strain SS9. The color of the arrows indicates the amino acid sequence identity between orthologs: white arrow indicates no homolog was found in the other set of ATPase or identity below 50%; grey arrow indicates 50–80% identity and black arrow indicates over 80% identity. The arrows are in proportion to the actual length of the genes and a scale bar of 0.5 kb is presented. Panel (**B**), the amplification of the intergenic region in ATPase-I (**top**) and ATPase-II (**bottom**) gene clusters. The 1 to 3 lanes for each intergenic region represent DNA, RNA and cDNA samples, respectively. The text below the image indicates the amplified region, for example *atpI*-*B*1 represent intergenic region between *atpI* and *atpB*1.

Genome analysis revealed that the ATPase locus located on chromosome 1 (PBPRA3603-3611, designated as ATPase-I locus) is 9120 bp in length and consists of nine genes. The other locus (designated as ATPase-II locus), located on chromosome 2 (PBPRB0130-0137) is 7300 bp in length and consists of eight genes (Figure 1A). Both sets of ATPase loci encode the eight obligatory subunits (subunit a, b, c, α, β, γ, δ and ε), with an additional *atpI* gene present upstream of *atpB* in ATPase-I. Among the obligatory components, the *atpA* and *atpD* genes encoding for the α and β subunits of the rotor were the most conserved, with over 80% amino acid identities. The *atpE* and *atpH* genes coding for subunits c and δ were the least conserved, with less than 50% amino acid identities between their homologs (Table S2). The expression pattern of the two ATPase gene clusters was analyzed. As shown in Figure 1B, the intergenic regions between adjacent genes of the ATPase-I locus can be amplified in the cDNA sample, suggesting it is expressed as an operon. In contrast, the intergenic regions in the ATPase-II gene cluster were not detected, indicating separated transcription or very low levels of gene transcription.

### 3.2. ATPase-I Acts As the Dominant ATPase under Conventional Cultural Condition

**Figure 2:**
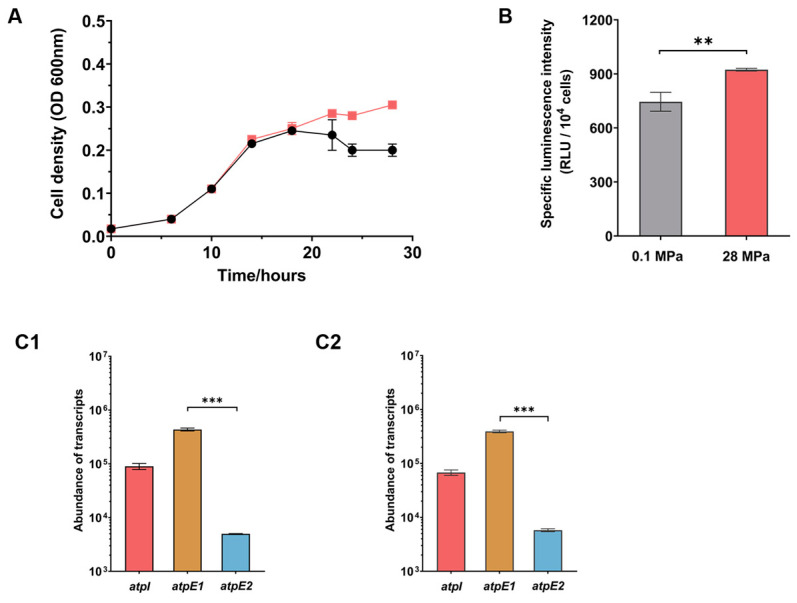
Growth curves, intracellular ATP level and expression levels of ATPase components in wild-type SS9R cells cultured under different pressure conditions in MB2216. Panel (**A**), growth curves of SS9R at 0.1 MPa and 28 MPa. The black lines represent growth at 0.1 MPa and the red lines represent growth at 28 MPa. Panel (**B**), intracellular ATP level in SS9R cells at exponential phase at 0.1 MPa and 28 MPa, the values show luminescence intensity per 10^4^ cells. Panel (**C1**,**C2**), abundance of *atpI*, *atpE*1 and *atpE*2 transcripts at 0.1 MPa (C1) and 28 MPa (C2), respectively. The statistical analysis of comparisons between two groups was performed using an unpaired-sample *t*-test: **, *p* < 0.01; ***, *p* < 0.001.

In order to elucidate the function of two sets of ATPase loci in strain SS9R, the growth and expression of select ATPase genes were examined under different pressures (atmospheric pressure [0.1 MPa] and the optimal growth pressure [28 MPa]). Growth experiments demonstrated that SS9R grown in Marine Broth 2216 (MB2216) medium had similar growth profiles at the different pressures until ~18 h after inoculation, at which point the fast-growing exponential phase continued at 28 MPa but stopped at 0.1 MPa, thus leading to a higher biomass in cultures at 28 MPa (Figure 2A and Table S3).

Cells cultured to exponential phase were collected for quantification of intracellular ATP and for RNA extraction. As shown in Figure 2B, SS9R cells grown at 28 MPa had a higher ATP level than cells at 0.1 MPa. Three ATPase genes were chosen to be analyzed: *atpI*, the unique gene present in the ATPase-I locus; and *atpE*1 and *atpE*2, each encoding the indispensable subunit c that forms part of the rotor, which is also one of the least conserved components among the eight obligatory subunits. Absolute quantitative RT-PCR analysis showed that at both 0.1 MPa and 28 MPa, the abundance of the *atpE*1 transcripts were approximately two orders of magnitude higher than its ortholog *atpE*2 in the ATPase-II locus, and around four times higher than that of *atpI* (Figure 2, panel C1 and C2). Taking the transcript levels of *atpE*1 and *atpE*2 to be indicators of the expression levels of the two ATPase systems, the results suggest that ATPase-I acts as the dominant ATPase under both pressure conditions. We then carried out a relative quantitative RT-PCR to evaluate influence of elevated pressure on two ATPase systems, and the results showed that all three genes examined transcribed constitutively under the two pressure conditions (Table S4).

### 3.3. Impairment in ATPase-I Results in Up-regulated Expression of ATPase-II

**Figure 3:**
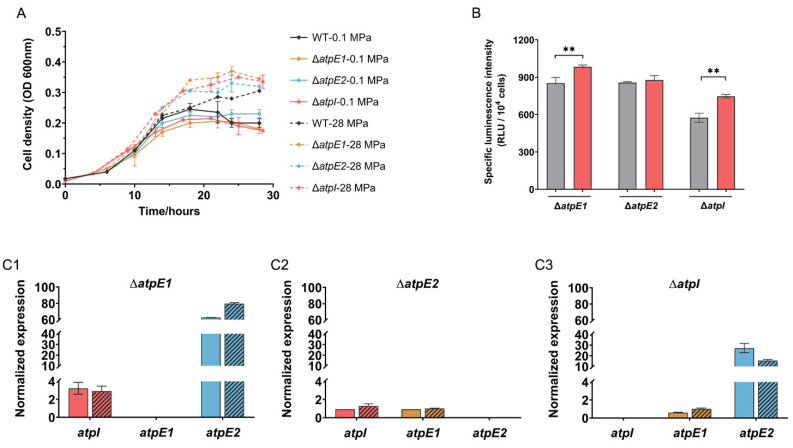
The growth curves, intracellular ATP level and expression of ATPase in Δ*atpE*1, Δ*atpE*2 and Δ*atpI* mutants. Panel (**A**), growth curves of wild-type and mutant strains at different pressures. Panel (**B**), specific intracellular ATP level in different mutants. The grey and red bars represent cultures at 0.1 MPa and 28 MPa, respectively. Panel (**C1**–**C3**), expression of *atpI*, *atpE*1 and *atpE*2 in the mutants relative to the wild-type strain. A value of 1 means no change from wild-type, whereas 100 mean a 100-fold increase in expression relative to wild type. The colored bars represent relative expression at 0.1 MPa, and colored bars with shadows represent relative expression at 28 MPa. Panel C1, C2 and C3 show the mutant of Δ*atpE*1, Δ*atpE*2 and Δ*atpI*, respectively. The statistical analysis of comparisons between two groups was performed using an unpaired-sample *t*-test: **, *p* < 0.01.

In order to clarify the function of each ATPase system, deletion mutants of Δ*atpE*1, Δ*atpE*2 and Δ*atpI* were constructed. All three mutant strains retained almost identical growth profiles to the wild-type strain at 0.1 MPa. At 28 MPa, a prolonged exponential phase was observed in the cultures of mutant strains, but their maximal growth rates (**μ_max_**) and final biomass were same as the wild-type strain (Figure 3A and Table S3). Resembling the wild-type strain, the mutant Δ*atpE*1 and Δ*atpI* grown at 28 MPa had a higher level of ATP. Meanwhile, Δ*atpE*2 cells had a similar abundance of ATP at 0.1 MPa and 28 MPa (Figure 3B).

We then examined the influence of an impaired ATPase system on the expression of other ATPase components. As anticipated, deletion of *atpE*2, which transcribed at a very low level in the wild-type strain, had no influence on the expression of *atpI* and *atpE*1 (Figure 3C2). On the contrary, the deletion of *atpE*1 greatly increased the expression of *atpE*2 by approximately 60-fold at 0.1 MPa, and 80-fold at 28 MPa, respectively, and slightly induced *atpI* by approximately 2-fold (Figure 3C1). Similarly, deletion of *atpI* induced *atpE*2 by 27-fold and 15-fold at 0.1 MPa and 28 MPa, respectively, but hardly affected the expression of *atpE*1 (Figure 3C3). As a result, the abundance of the *atpE*2 transcripts was comparable to that of *atpE*1 in the Δ*atpI* mutant (Figure S1). The disruption of ATPase-I locus up-regulated expression of genes from ATPase-II locus suggested that the two systems are regulated correlatively. Moreover, judging by the growth profile and level of ATP, induction of ATPase-II genes is capable of overcoming the effects of mutation in the ATPase-I locus. It is thus speculated that the two ATPase systems are functionally redundant and a single ATPase system alone is sufficient to fulfill the growth requirements of SS9R.

### 3.4. SS9R Cells Had a More Pronounced Piezophilic Phenotype When Cultivated in MG Medium

**Figure 4:**
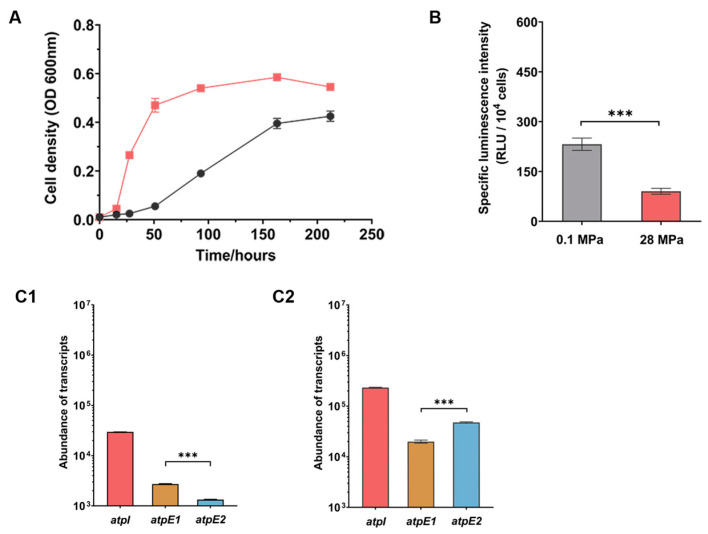
Growth curves, ATP level and expression of ATPase genes in SS9R wild-type strain cultured in MG medium. Panel (**A**), growth curve of SS9R at 0.1 MPa and 28 MPa. The black lines represent growth at 0.1 MPa and the red lines represent growth at 28 MPa. Panel (**B**), intracellular ATP level in S99R cells at exponential phase at 0.1 MPa and 28 MPa; the values show luminescence intensity per 10^4^ cells. Panel (**C1**,**C2**), abundance of *atpI*, *atpE*1 and *atpE*2 transcripts at 0.1 MPa (**C1**) and 28 MPa (**C2**), respectively. The statistical analysis of comparisons between two groups was performed using an unpaired-sample *t*-test: ***, *p* < 0.001.

The marine broth 2216 medium is conventionally used for the cultivation of the SS9R strain, but the complex composition makes this medium less ideal for metabolism analysis. We then cultured the SS9R cells in a defined medium using glucose as a carbon source and NH_4_Cl as a nitrogen source (MG medium). To our surprise, the growth of SS9R at 0.1 MPa and 28 MPa were remarkably different from the beginning of exponential phase. Cultures at 28 MPa had a maximal specific growth rate (μ_max_) two times higher than that of cultures at 0.1 MPa, resulting in a more typical and more pronounced piezophilic phenotype than the cultures in MB2216 medium (Figure 4A and Table S3, versus Figure 2A). Quantification of intracellular ATP showed that ATP levels varied with pressures, but exhibited a reversed trend compared to the cells grown in MB2216: cells grown at 0.1 MPa had more abundant ATP than those grown at 28 MPa (Figure 4B). In addition to that, intracellular ATP decreased by over 2-fold at 28 MPa in cells grown in MG medium (luminescent intensity of 90 RLU/10^4^ cells at 28 MPa versus 232 RLU/10^4^ cells at 0.1 MPa), more significant than the changes in cells grown in MB2216 (luminescent intensity of 924 RLU/10^4^ cells at 28 MPa versus 745 RLU/10^4^ cells at 0.1 MPa), indicating the cells are more sensitive to changing of pressure under this cultural condition.

### 3.5. ATPase-II Is Preferred When Cultivated in MG Medium

Quantification of ATPase gene transcript levels showed that, under both pressure conditions, the *atpI* gene had the highest number of transcripts, approximately one order of magnitude over those of the other two genes examined. The transcriptional level of the two orthologs of *atpE*: *atpE*1 and *atpE*2, were similar to one another, although *atpE*1 transcripts had a slightly higher abundance at 0.1 MPa and *atpE*2 transcripts were more abundant at 28 MPa (Figure 4C1,C2). Unlike cells grown in MB2216, expression of the ATPase genes were affected by changing the pressure from 0.1 MPa to 28 MPa. The expression of *atpI* and *atpE*1 were repressed at 28 MPa by 4- and 2-fold, respectively, while the expression of *atpE*2 was unaffected (Table S4).

The effect of culture medium on the expression of ATPase was also examined at 0.1 MPa and 28 MPa. Compared to cells grown in MB2216, *atpI* and *atpE*2 were induced about 20- and 8-fold, respectively, in MG medium at 0.1 MPa, and around 4- and 9-fold at 28 MPa, respectively, while the expression of *atpE*1 was repressed in the MG medium at 28 MPa (Table 1). This observation explained the expression profile obtained by absolute quantification, in which *atpE*1 had more transcripts than *atpE*2 when cultivated in MB2216, but comparable abundance in MG medium (Figure 2 and Figure 4).

Collectively, culture medium imposed greater influence on the expression of ATPase than increased pressure from 0.1 MPa to 28 MPa, and the two ATPase systems reacted differently to these factors. ATPase-II seems to be more sensitive to a change of culture medium, and is preferred in cells grown in MG medium, indicating a possible contribution of ATPase-II to the pronounced piezophilic phenotype.

### 3.6. Mutated ATPase-I Improved Growth at 0.1 MPa in MG Medium

**Figure 5:**
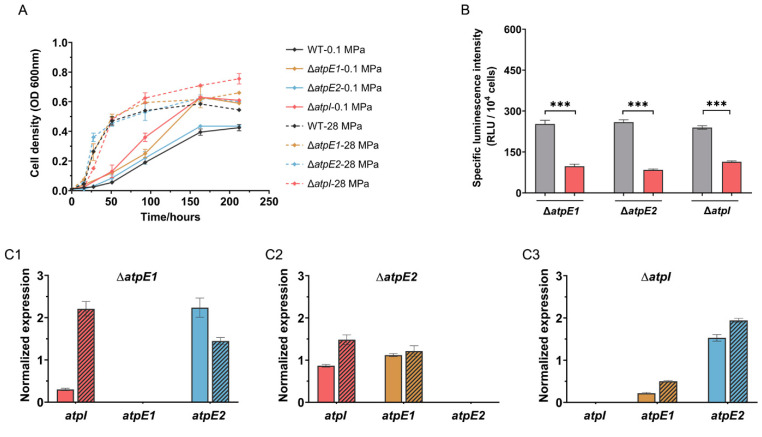
The growth curves, intracellular ATP level and expression of ATPase in Δ*atpE*1, Δ*atpE*2 and Δ*atpI* mutants grown in MG medium. Panel (**A**), growth curves of wild-type and mutant strains at different pressures. Panel (**B**), specific intracellular ATP level in different mutants. The grey and red bars represent cultures at 0.1 MPa and 28 MPa, respectively. Panel (**C1**–**C3**), expression of *atpI*, *atpE*1 and *atpE*2 in the mutants relative to the wild-type strain. The colored bars represent relative expression at 0.1 MPa, and colored bars with shadows represent relative expression at 28 MPa. Panels C1, C2 and C3 show the mutant of Δ*atpE*1, Δ*atpE*2 and Δ*atpI*, respectively. The statistical analysis of comparisons between two groups was performed using an unpaired-sample *t*-test: ***, *p* < 0.001.

In order to understand the relationship of two ATPase systems when the cells were cultivated in MG medium, we further examined the Δ*atpE*1, Δ*atpE*2 and Δ*atpI* mutants under this culture condition. The growth profile of Δ*atpE*2 resembled the wild-type strain at both atmospheric and elevated pressures, while the growth of Δ*atpE*1 and Δ*atpI* at 0.1 MPa were clearly promoted and had significantly higher biomass than the wild-type strain (Figure 5A and Table S3). The intracellular ATP level was not affected by mutation of either ATPase system under this culture condition, similar to the wild-type strain; all three mutants had a significantly higher level of ATP at 0.1 MPa (Figure 5B).

Unlike cells cultivated in MB2216, the expression of ATPase genes were hardly affected by mutation of *atpE*1, *atpE*2 or *atpI* when growing in MG medium. The exceptions were the transcript abundances of *atpI* in the Δ*atpE*1 mutant and *atpE*1 in the Δ*atpI* mutant at 0.1 MPa, which were repressed approximately by 5-fold (Figure 5C1–C3). Furthermore, as anticipated, the abundance of the three gene transcripts at 28 MPa were similar to the wild-type strain under this culture condition (Figure S2).

## 4. Discussion

In this study, we showed that under conventional culture conditions (MB2216 medium), ATPase-I encoded on chromosome 1 played a dominant role and the transcript abundance of ATPase-II components was a hundred times less and hardly detectable by routine RT-PCR. This is consistent with transcriptome analysis showing that genes on chromosome 1 are more actively transcribed while genes on chromosome 2 are poorly expressed [21]. Further analyses of the mutants showed that, as anticipated, deletion of the minor ATPase-II had little influence on growth and intracellular ATP level. On the other hand, deletion of the ATPase-I component (either *atpI* or *atpE*1) induced expression of ATPase-II (*atpE*2) to a level close to *atpE*1 in the wild-type strain, and the growth and intracellular ATP level were not affected in the mutants. This suggests that despite differences in gene composition and divergence among subunits (notably subunits c and δ), the two ATPase systems are substitutable, and a single ATPase would be sufficient to fulfill the requirement for cellular growth in this medium. The observation that the presence of a functional ATPase-I system has a repression effect on the expression of the ATPase-II locus suggests that the two ATPase systems are coordinately regulated. However, the mechanism underlying this control remains unknown. Whether functional ATPase-I represses expression of ATPase-II in a direct manner or through any global regulator will require further investigation.

When the cells were grown in a defined minimal medium supplemented with glucose, the situation was quite different. The two ATPase loci express similar transcript abundances, and disruption of either ATPase system had little effect on the expression of the other one or the ATP level. It is thus speculated that, relative to cultures in MB2216, the contribution of ATPase-II is increased under this culture condition. It should be noted that apart from oxidative phosphorylation, ATP could also be generated through substrate-level phosphorylation, which does not require the involvement of membrane ATPase. We cannot rule out the possibility that ATP was, at least partially, generated through glycolysis and ATPase may be dispensable under this culture condition. In fact, a similar observation was made for the aforementioned deep-sea *Shewanella* strain WP3. The genome-scale modeling study suggested that the deletion of genes responsible for substrate-level phosphorylation decreased biomass flux, while deletion of ATPase had little influence, indicating a more important role of substrate-level phosphorylation instead of oxidative phosphorylation during anaerobic growth [30]. It would be helpful to obtain a comprehensive understanding of the energy metabolism profile in SS9 cells by means of transcriptomic or proteomic analysis.

It has been previously reported that microbial tolerance to HHP could be influenced by energy metabolism. Supplementation of TMAO in the culture medium of the deep-sea strain *Vibrio fluvialis* QY27 improves its growth at high pressure by inducing expression of TMAO reductase and accelerating the anaerobic TMAO respiration [31]. In this study a great difference in high-pressure growth was observed when deep-sea *P. profundum* SS9R was grown in different media. The wild-type strain grew similarly at 0.1 MPa and 28 MPa in the MB2216 medium, but had a remarkably accelerated growth and pronounced piezophilic phenotype at its optimum pressure when grown in the MG medium. The MB2216 medium has a complex composition, and may provide electron acceptors and substrates for diverse respiration and fermentation pathways. However, the cells grown in MG medium may generate energy through limited pathways and mostly rely on glucose fermentation. A proteomic analysis of SS9 revealed that proteins involved in the glycolysis pathway were up-regulated at 28 MPa, while proteins involved in the oxidative phosphorylation pathway had higher abundance at 0.1 MPa [32], which may explain the more significant piezophilic growth phenotype of *P. profundum* SS9 grown in the MG medium. Yet, with the limited information we have at present, the mechanism of glucose-improved high-pressure growth remains unknown. It would be worthwhile to carry out a more systematic analysis of the expression of energy metabolism-related genes, and the abundance and changing profiles of metabolites at different pressure conditions to examine which metabolic processes are activated under this culture condition.

We have conducted a preliminary genomic survey on the number and type of ATPases in the genus of *Photobacterium* on the Integrated Microbial Genomes (IMG) platform (https://img.jgi.doe.gov/) (accessed on 30 January 2023). In total 164 *Photobacterium* genomes consisting of 34 species were analyzed. Among them, approximately 80% of the *Photobacterium* strains (130 strains from 18 species) encode two sets of ATPase loci, just as is the case for deep-sea *P. profundum* strain SS9, and 31 strains from 15 species have a single ATPase, which in all cases is most closely related to the ATPase-I locus present in SS9. In another three strains, three copies of ATPase were identified, including two copies more closely related to ATPase-I. It therefore seems that redundant ATPase systems is the rule rather than the exception in *Photobacterium*. The two ATPase loci identified in strain SS9 are located on different chromosomes. Studies of *Vibrio* and *Photobacterium* suggested that the secondary small chromosome contains more lineage-specific genes and unknown genes that evolve more rapidly than those encoded on the large chromosome, and these genes might have a peculiar function under specific niches or conditions [33,34]. Taking into consideration the different free-living and symbiotic life-styles and universal distribution of *Photobacterium* species in seawater, marine sediments, saline lake water, as well as in association with marine animals such as fishes, it is possible that the two copies of ATPase might have other physiological roles outside of adaptation to elevated pressure.

One of the major differences between the two ATPase systems in the SS9 strain is the absence of AtpI, the least studied subunit of the F-ATPase system, in ATPase-II. The AtpI (also known as UncI) is a small inner-membrane protein that functions as a chaperone to mediate the assembly of the c-ring. It can hardly be detected in *E. coli* cells and its disruption has little influence on the function of ATPase. However, recent studies showed that it plays an essential role in the assembly of a functional Na^+^ ATPase [35,36,37]. The identity of amino acid sequence between AtpI from strain SS9 and *E. coli* K-12 was 30.25%, much lower than the value of other ATPase components (ranging from 50.75% to 82.26%). It was thus wondered if it plays a unique role that distinguishes the two sets of ATPases. We noticed that the Δ*atpI* mutant behaves almost identically to Δ*atpE*1 with respect to the induction of *atpE*2 expression and improvement of growth in MG medium. This indicates that, like AtpE1, AtpI is also indispensable for the function of ATPase-I, and its specific role in the assembly of ATPase-I requires further investigation.

## 5. Conclusions

In this study, we showed that the deep-sea bacterium *Photobacterium profundum* SS9 exhibited a more pronounced piezophilic phenotype when grown in minimal medium supplemented with glucose than in the routinely used complex MB2216 medium. In both culture media, elevated pressure influenced intracellular ATP level, but with opposite effects. The two sets of ATPase encoded by SS9 are correlatively regulated and functionally substitutable. When the cells were grown in MB2216, disruption of the dominant ATPase-I induced the expression of ATPase-II, which complements the growth phenotype. When the cells were grown in MG medium, the abundance of ATPase-II increased, especially at elevated pressure when cells had the lowest ATP level among all conditions tested. Collectively, our results demonstrated the differences and relationship between the two ATPase systems in a piezophilic bacterium and expanded the understanding of the involvement of energy metabolism in high-pressure adaptation.

## Figures and Tables

**Table 1:** The influence of cultural medium on the expression of ATPase genes in SS9R and the mutant strains.

	SS9R		Δ*atpE*1		Δ*atpE*2		Δ *atpI*	
	0.1 MPa	28 MPa	0.1 MPa	28 MPa	0.1 MPa	28 MPa	0.1 MPa	28 MPa
*atpI*	20.13 ± 1.06 ^#,^***	3.58 ± 0.19 ***	1.89 ± 0.18 *	2.75 ± 0.21 ***	6.77 ± 0.23 ***	4.18 ± 0.32 ***	-	-
*atpE*1	0.52 ± 0.05 **	0.23 ± 0.01 ***	-	-	0.62 ± 0.02 ***	0.27 ± 0.03 ***	0.19 ± 0.02 **	0.11 ± 0.00 ***
*atpE*2	8.48 ± 1.32 ***	9.08 ± 0.97 ***	0.46 ± 0.15	0.16 ± 0.01 ***	-	-	0.48 ± 0.02 **	1.14 ± 0.03

^#^ The values presented are relative gene expression in MG medium relative to MB2216 medium. All data are shown as mean ± standard deviation. The statistical analysis of comparisons between two groups was performed using an unpaired-sample *t*-test, and a two-tailed *p* < 0.05 was considered significant: *, *p* < 0.05; **, *p* < 0.01; ***,*p* < 0.001.

## Data Availability

All the relevant data related to this study are presented in the manuscript.

## Supplementary Materials

The following supporting information can be downloaded at: https://www.mdpi.com/article/10.3390/microorganisms11030637/s1, Table S1: Primers used in this study. Table S2: The genetic characterization of ATPase components in SS9 strain. Table S3: The growth characteristics of wild-type and mutant strains in MB2216 and MG media. Table S4: Effect of pressure on the expression of ATPase genes. Figure S1: Abundance of ATPase genes in mutant strains grown in MB2216 medium. Figure S2: Abundance of ATPase genes in mutant strains grown in MG medium.

## References

[1] Bartlett D.H. (2002). Pressure effects on in vivo microbial processes. Biochim. Biophys. Acta.

[2] Gross M., Jaenicke R. (1990). Pressure-induced dissociation of tight couple ribosomes. FEBS Lett..

[3] Balny C., Masson P., Heremans K. (2002). High pressure effects on biological macromolecules: From structural changes to alteration of cellular processes. Biochim. Biophys. Acta.

[4] Kato C., Nogi Y., Arakawa S. (2008). Isolation, Cultivation, and Diversity of Deep-Sea Piezophiles. High-Pressure Microbiology.

[5] Amrani A., Bergon A., Holota H., Tamburini C., Garel M., Ollivier B., Imbert J., Dolla A., Pradel N. (2014). Transcriptomics Reveal Several Gene Expression Patterns in the Piezophile Desulfovibrio hydrothermalis in Response to Hydrostatic Pressure. PLoS ONE.

[6] Amrani A., van Helden J., Bergon A., Aouane A., Ben Hania W., Tamburini C., Loriod B., Imbert J., Ollivier B., Pradel N. (2016). Deciphering the adaptation strategies of Desulfovibrio piezophilus to hydrostatic pressure through metabolic and transcriptional analyses. Environ. Microbiol. Rep..

[7] Ohke Y., Sakoda A., Kato C., Sambongi Y., Kawamoto J., Kurihara T., Tamegai H. (2013). Regulation of cytochrome c- and quinol oxidases, and piezotolerance of their activities in the deep-sea piezophile Shewanella violacea DSS12 in response to growth conditions. Biosci. Biotechnol. Biochem..

[8] Yamada M., Nakasone K., Tamegai H., Kato C., Usami R., Horikoshi K. (2000). Pressure regulation of soluble cytochromes c in a deep-sea piezophilic bacterium, Shewanella violacea. J. Bacteriol..

[9] Tamegai H., Kawano H., Ishii A., Chikuma S., Nakasone K., Kato C. (2005). Pressure-regulated biosynthesis of cytochrome bd in piezo- and psychrophilic deep-sea bacterium Shewanella violacea DSS12. Extremophiles.

[10] Kilic V., Kilic G.A., Kutlu H.M., Martinez-Espinosa R.M. (2017). Nitrate reduction in Haloferax alexandrinus: The case of assimilatory nitrate reductase. Extremophiles.

[11] Chen Y., Wang F.P., Xu J., Mehmood M.A., Xiao X. (2011). Physiological and evolutionary studies of NAP systems in Shewanella piezotolerans WP3. ISME J..

[12] Li X.G., Zhang W.J., Xiao X., Jian H.H., Jiang T., Tang H.Z., Qi X.Q., Wu L.F. (2018). Pressure-Regulated Gene Expression and Enzymatic Activity o the Two Periplasmic Nitrate Reductases in the Deep-Sea Bacterium Shewanella piezotolerans WP3. Front. Microbiol..

[13] Xiong L., Jian H.H., Zhang Y.X., Xiao X. (2016). The Two Sets of DMSO Respiratory Systems of Shewanella piezotolerans WP3 Are Involved in Deep Sea Environmental Adaptation. Front. Microbiol..

[14] Xiong L., Jian H.H., Xiao X. (2017). Deep-Sea Bacterium Shewanella piezotolerans WP3 Has Two Dimethyl Sulfoxide Reductases in Distinct Subcellular Locations. Appl. Environ. Microbiol..

[15] Brandt K., Muller D.B., Hoffmann J., Hubert C., Brutschy B., Deckers-Hebestreit G., Muller V. (2013). Functional production of the Na+ F1F(O) ATP synthase from Acetobacterium woodii in Escherichia coli requires the native AtpI. J. Bioenerg. Biomembr..

[16] Dreyfus G., Guimaraes-Motta H., Silva J.L. (1988). Effect of hydrostatic pressure on the mitochondrial ATP synthase. Biochem. -Us.

[17] Souza M.O., Creczynski-Pasa T.B., Scofano H.M., Graber P., Mignaco J.A. (2004). High hydrostatic pressure perturbs the interactions between CF0F1 subunits and induces a dual effect on activity. Int. J. Biochem. Cell Biol..

[18] Okuno D., Nishiyama M., Noji H. (2014). Single-Molecule Analysis of the Rotation of F1-ATPase under High Hydrostatic Pressure. Biophys. J..

[19] Nogi Y., Masui N., Kato C. (1998). Photobacterium profundum sp. nov., a new, moderately barophilic bacterial species isolated from a deep-sea sediment. Extremophiles.

[20] DeLong E.F., Franks D.G., Yayanos A.A. (1997). Evolutionary relationships of cultivated psychrophilic and barophilic deep-sea bacteria. Appl. Environ. Microb..

[21] Vezzi A., Campanaro S., D’Angelo M., Simonato F., Vitulo N., Lauro F.M., Cestaro A., Malacrida G., Simionati B., Cannata N. (2005). Life at depth: Photobacterium profundum genome sequence and expression analysis. Science.

[22] Lauro F.M., Eloe E.A., Liverani N., Bertoloni G., Bartlett D.H. (2005). Conjugal vectors for cloning, expression, and insertional mutagenesis in gram-negative bacteria. Biotechniques.

[23] Welch T.J., Bartlett D.H. (1998). Identification of a regulatory protein required for pressure-responsive gene expression in the deep-sea bacterium Photobacterium species strain SS9. Mol. Microbiol..

[24] Eloe E.A., Lauro F.M., Vogel R.F., Bartlett D.H. (2008). The deep-sea bacterium Photobacterium profundum SS9 utilizes separate flagellar systems for swimming and swarming under high-pressure conditions. Appl. Environ. Microbiol..

[25] El-Hajj Z.W., Allcock D., Tryfona T., Lauro F.M., Sawyer L., Bartlett D.H., Ferguson G.P. (2010). Insights into piezophily from genetic studies on the deep-sea bacterium, Photobacterium profundum SS9. Ann. N. Y. Acad. Sci..

[26] Campanaro S., Pascale F.D., Telatin A., Schiavon R., Bartlett D.H., Valle G. (2012). The transcriptional landscape of the deep-sea bacterium Photobacterium profundum in both a toxR mutant and its parental strain. BMC Genomics.

[27] Tamegai H., Nishikawa S., Haga M., Bartlett D.H. (2012). The respiratory system of the piezophile Photobacterium profundum SS9 grown under various pressures. Biosci. Biotechnol. Biochem..

[28] Zhang S.D., Santini C.L., Zhang W.J., Barbe V., Mangenot S., Guyomar C., Garel M., Chen H.T., Li X.G., Yin Q.J. (2016). Genomic and physiological analysis reveals versatile metabolic capacity of deep-sea Photobacterium phosphoreum ANT-2200. Extremophiles.

[29] Chi E., Bartlett D.H. (1993). Use of a reporter gene to follow high-pressure signal transduction in the deep-sea bacterium Photobacterium sp. strain SS9. J. Bacteriol..

[30] Dufault-Thompson K., Jian H., Cheng R., Li J., Wang F., Zhang Y. (2017). A Genome-Scale Model of Shewanella piezotolerans Simulates Mechanisms of Metabolic Diversity and Energy Conservation. Msystems.

[31] Yin Q.J., Zhang W.J., Qi X.Q., Zhang S.D., Jiang T., Li X.G., Chen Y., Santini C.L., Zhou H., Chou I.M. (2018). High Hydrostatic Pressure Inducible Trimethylamine N-Oxide Reductase Improves the Pressure Tolerance of Piezosensitive Bacteria Vibrio fluvialis. Front. Microbiol..

[32] Le Bihan T., Rayner J., Roy M.M., Spagnolo L. (2013). Photobacterium profundum under pressure: A MS-based label-free quantitative proteomics study. PLoS ONE.

[33] Urbanczyk H., Ast J.C., Dunlap P.V. (2011). Phylogeny, genomics, and symbiosis of Photobacterium. FEMS Microbiol. Rev..

[34] Cooper V.S., Vohr S.H., Wrocklage S.C., Hatcher P.J. (2010). Why genes evolve faster on secondary chromosomes in bacteria. PLoS Comput. Biol..

[35] Suzuki T., Ozaki Y., Sone N., Feniouk B.A., Yoshida M. (2007). The product of uncI gene in F1Fo-ATP synthase operon plays a chaperone-like role to assist c-ring assembly. Proc. Natl. Acad. Sci. USA.

[36] Gay N.J. (1984). Construction and characterization of an Escherichia coli strain with a uncI mutation. J. Bacteriol..

[37] Ozaki Y., Suzuki T., Kuruma Y., Ueda T., Yoshida M. (2008). UncI protein can mediate ring-assembly of c-subunits of FoF1-ATP synthase in vitro. Biochem. Biophys. Res. Commun..

