# Supplementary material for: Piezophilic Phenotype Is Growth Condition Dependent and Correlated with the Regulation of Two Sets of ATPase in Deep-Sea Piezophilic Bacterium Photobacterium profundum SS9
Source: Microorganisms. 2023 Mar 2;11(3):637. doi: 10.3390/microorganisms11030637 (PMC10054830; doi:10.3390/microorganisms11030637)
Supplement: Supplementary file 1 [file microorganisms-11-00637-s001.zip › microorganisms-2243501-supplementary.pdf]

# Piezophilic phenotype is growth condition dependent and correlated with the regulation of two sets of ATPase in deep-sea piezophilic bacterium *Photobacterium profundum* SS9

An-qi Li[1,2,3], Wei-jia Zhang[3,4,5*], Xue-gong Li[3,4,5], Xu-chong Bao[2,3], Xiao-qing Qi[3,4,5], Long-Fei Wu[4,6] and Douglas Bartlett[7]

[1] Institute of Oceanology, Chinese Academy of Sciences, Qingdao 266071, China
[2] University of Chinese Academy of Sciences, Beijing 101408, China
[3] Laboratory of Deep-Sea Microbial Cell Biology, Institute of Deep-sea Science and Engineering, Chinese Academy of Sciences, Sanya 572000, China
[4] International Associated Laboratory of Evolution and Development of Magnetotactic Multicellular Organisms, CNRS-Marseille/CAS-Sanya, Sanya 572000, China
[5] Institution of Deep-sea Life Sciences, IDSSE-BGI, Hainan Deep-sea Technology Laboratory, Sanya 572000, China
[6] LCB, Aix Marseille University and CNRS, 13402 Marseille, France
[7] Marine Biology Research Division, Scripps Institution of Oceanography, University of California San Diego, La Jolla, CA 92093, USA

* Correspondence: Dr. Wei-Jia Zhang; E-mail: wzhang@idsse.ac.cn; Tel: +86 898 8821 1771. Laboratory of Deep-Sea microbial cell biology, Institute of Deep-sea Science and Engineering, Chinese Academy of Sciences, Sanya, Hainan 572000, China.

**Keywords:** Deep-sea bacterium; high hydrostatic pressure; piezophilic, ATPase; energy metabolism

**Table S1.** Primers used in this study

| Name | Sequence (5′-3′) | Description |
|---|---|---|
| I-up-F | CGAGCTCTATTTAGCCCTCGCTGGTG | Deletion of *atpI* |
| Fusion-I-R | CAGCCATAACTCATCGTATTCGTTCCTCG | Deletion of *atpI* |
| Fusion-I-F | CGAGGAACGAATACGATGAGTTATGGCTG | Deletion of *atpI* |
| I-down-R | TGCTCTAGAGTACCAAGGGAAGCAAG | Deletion of *atpI* |
| SE1-up-F | CCCCCGGGAGTTATCTTATTTGCCATTGTTTAC | Deletion of *atpE1* |
| SE1-up-R | TGCTCTAGATATCTCCAGTTTATAGTTGTCGGC | Deletion of *atpE1* |
| SE1-d-F | TGCTCTAGATCAATAGCTGTTTTAACCTTGTGG | Deletion of *atpE1* |
| SE1-d-R | TCCCCGCGGCACTGCAATTCAGCTTAACTTTACG | Deletion of *atpE1* |
| SE2-up-F | CCCCCGGGAATAAGAGTCACTAGAACGGTCAAG | Deletion of *atpE2* |
| SE2-up-R | TGCTCTAGAAATTTTTTATAAATTAAATTAACG | Deletion of *atpE2* |
| SE2-d-F | TGCTCTAGATAAATGACTACTGCGGTGATACAG | Deletion of *atpE2* |
| SE2-d-R | TCCCCGCGGACTCAACAGTGCGAGAAAG | Deletion of *atpE2* |
| Q-I-F | TTAATCGGTGGTGGCATC | RT-PCR analysis |
| Q-I-R | GGTCAGTTTGAGGGGAAC | RT-PCR analysis |
| Q-E1-F | GTTTCGCTCTTCTTGGTGG | RT-PCR analysis |
| Q-E1-R | AGCTAGCTGGGCAACAAATG | RT-PCR analysis |
| Q-E2-F | ACGTTGCTGGCGCAC | RT-PCR analysis |
| Q-E2-R | TACCAACACCGATCATTGG | RT-PCR analysis |
| G3PDH-F | CGGCCTTCTAACAGGTATGG | RT-PCR analysis |
| G3PDH-R | AGTTAGCGCAACACCAGC | RT-PCR analysis |

**Table S2.** The genetic characterization of ATPase components in SS9 strain

| | ATPase-I | | ATPase-II | | Gene product | Similarity in amino acid sequences (%) |
|---|---|---|---|---|---|---|
| | Gene locus | Length (amino acids) | Gene locus | Length (amino acids) | | |
| ***atpI*** | PBPRA3611 | 129 | - | - | Subunit I | - |
| ***atpB*** | PBPRA3610 | 262 | PBPRB0130 | 255 | Subunit a | 55.73 |
| ***atpE*** | PBPRA3609 | 86 | PBPRB0131 | 82 | Subunit c | 44.74 |
| ***atpF*** | PBPRA3608 | 156 | PBPRB0132 | 134 | Subunit b | 57.48 |
| ***atpH*** | PBPRA3607 | 177 | PBPRB0133 | 182 | Subunit δ | 44.51 |
| ***atpA*** | PBPRA3606 | 513 | PBPRB0134 | 513 | Subunit α | 82.85 |
| ***atpG*** | PBPRA3605 | 288 | PBPRB0135 | 291 | Subunit γ | 68.49 |
| ***atpD*** | PBPRA3604 | 468 | PBPRB0136 | 461 | Subunit β | 83.12 |
| ***atpC*** | PBPRA3603 | 140 | PBPRB0137 | 147 | Subunit ε | 58.39 |

**Table S3.** The growth characteristics of wild-type and mutant strains in MB2216 and MG media

| | MB2216 medium | | | | MG medium | | | |
|---|---|---|---|---|---|---|---|---|
| | $\mu_{max}$ | | Biomass ($OD_{600nm}$) | | $\mu_{max}$ | | Biomass ($OD_{600nm}$) | |
| | **0.1 MPa** | **28 MPa** | **0.1 MPa** | **28 MPa** | **0.1 MPa** | **28 MPa** | **0.1 MPa** | **28 MPa** |
| **WT** | 0.25 ± 0.00 | 0.25 ± 0.00 | 0.19 ± 0.01 | 0.30 ± 0.02 | 0.04 ± 0.00 | 0.10 ± 0.01 | 0.42 ± 0.01 | 0.54 ± 0.01 |
| **Δ*atpI*** | 0.26 ± 0.03 | 0.31 ± 0.02 | 0.18 ± 0.00 | 0.35 ± 0.00 | 0.03 ± 0.00 | 0.12 ± 0.01 | 0.61 ± 0.01 | 0.75 ± 0.02 |
| **Δ*atpE1*** | 0.22 ± 0.01 | 0.28 ± 0.00 | 0.15 ± 0.01 | 0.34 ± 0.01 | 0.02 ± 0.00 | 0.08 ± 0.00 | 0.59 ± 0.00 | 0.66 ± 0.01 |
| **Δ*atpE2*** | 0.29 ± 0.01 | 0.33 ± 0.00 | 0.20 ± 0.01 | 0.30 ± 0.01 | 0.03 ± 0.01 | 0.19 ± 0.01 | 0.43 ± 0.01 | 0.60 ± 0.01 |

**Table S4.** Effect of pressure on the expression of ATPase genes

| | | WT | *ΔatpI* | *ΔatpE1* | *ΔatpE2* |
|---|---|---|---|---|---|
| MB2216 | *atpI* | 1.47 ± 0.08[#, **] | - | 1.35 ± 0.25 | 0.74 ± 0.13[***] |
| | *atpE1* | 0.90 ± 0.07 | 1.50 ± 0.13[**] | - | 0.97 ± 0.02 |
| | *atpE2* | 1.22 ± 0.11 | 0.81 ± 0.10[*] | 1.35 ± 0.20[*] | - |
| MG | *atpI* | 0.26 ± 0.01[***] | - | 1.95 ± 0.16[**] | 0.45 ± 0.04[***] |
| | *atpE1* | 0.40 ± 0.01[***] | 0.92 ± 0.03 | - | 0.43 ± 0.05[***] |
| | *atpE2* | 1.32 ± 0.14 | 1.68 ± 0.04[***] | 0.86 ± 0.05 | - |

[#] The values show the relative expression at 28 MPa normalized to that at 0.1 MPa.

All data are shown as mean ± standard deviation. The statistical analysis of comparisons between two groups was performed using an unpaired-sample *t* test, a two-tailed $p < 0.05$ was considered significant, *, $p<0.05$; **, $p<0.01$; ***, $p<0.001$.

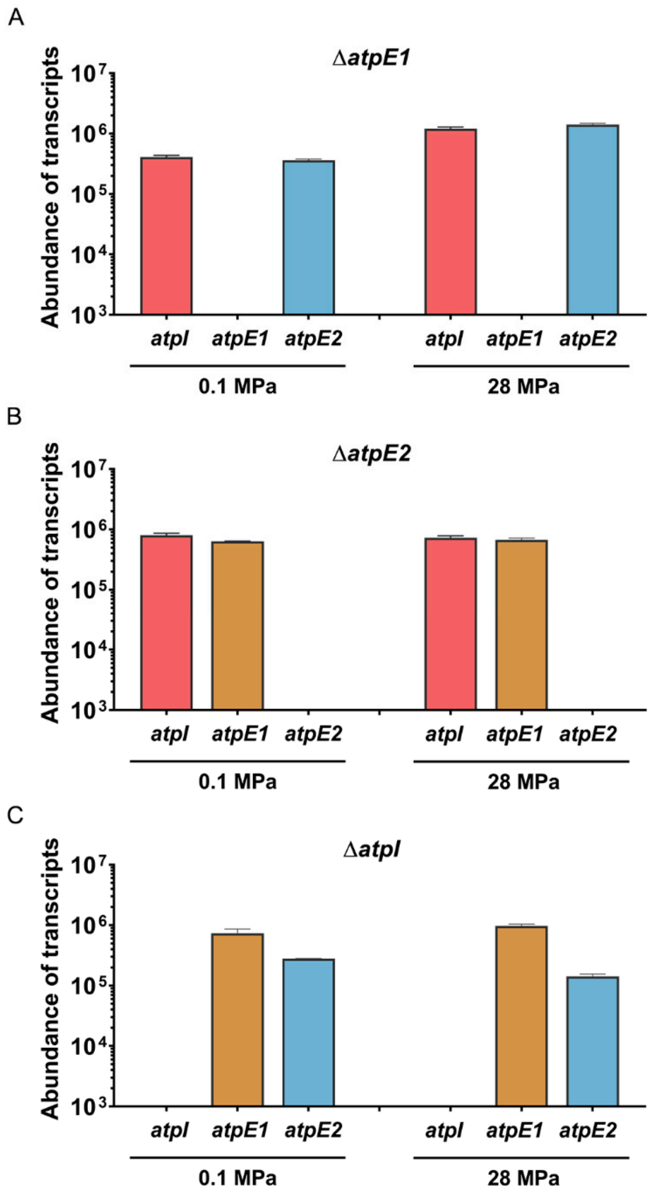


**Figure S1.** Abundance of ATPase gene transcripts in mutant strains grown in MB2216 medium. Panel A, B and C show the quantification of ATPase genes in Δ*atpE1*, Δ*atpE2* and Δ*atpI* mutants, respectively. The genes analyzed and pressure condition are marked below the bars.

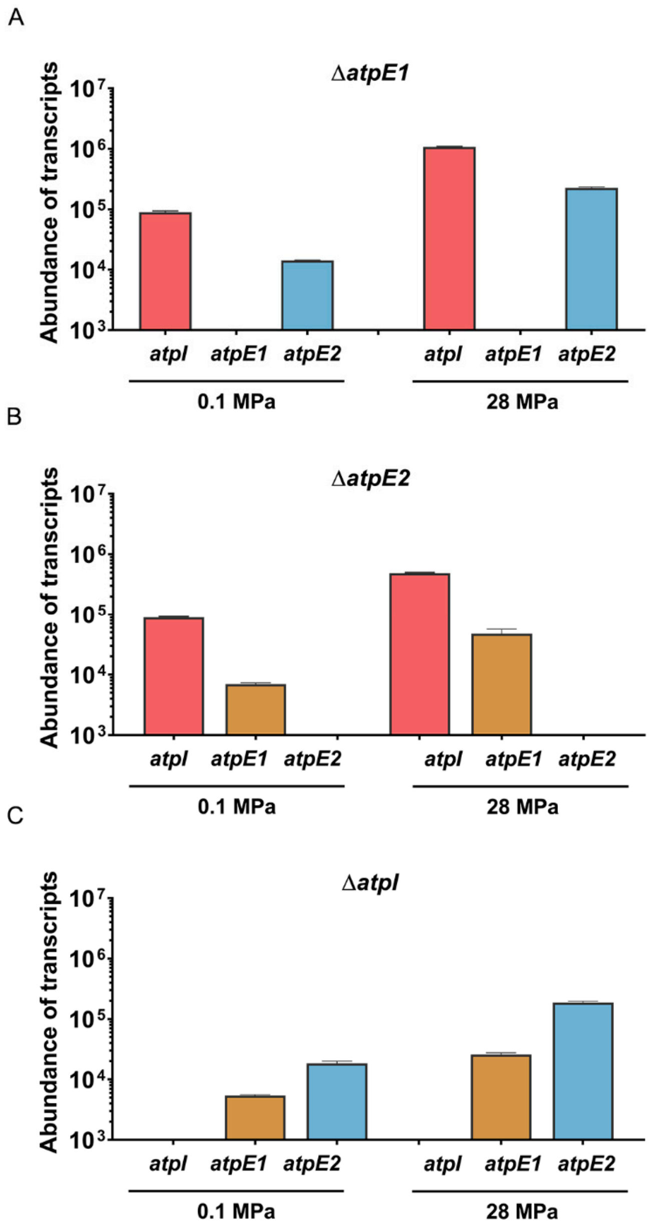


**Figure S2.** Abundance of ATPase gene transcripts in mutant strains grown in MG medium. Panel A, B and C show the quantification of ATPase genes in Δ*atpE1*, Δ*atpE2* and Δ*atpI* mutants, respectively. The genes analyzed and pressure condition are marked below the bars.
